# Molecular mechanism: the human dopamine transporter histidine 547 regulates basal and HIV-1 Tat protein-inhibited dopamine transport

**DOI:** 10.1038/srep39048

**Published:** 2016-12-14

**Authors:** Pamela M. Quizon, Wei-Lun Sun, Yaxia Yuan, Narasimha M. Midde, Chang-Guo Zhan, Jun Zhu

**Affiliations:** 1 Department of Drug Discovery and Biomedical Sciences, South Carolina College of Pharmacy, University of South Carolina, Columbia, SC, USA; 2 Molecular Modeling and Biopharmaceutical Center, College of Pharmacy, University of Kentucky, Lexington, KY, USA; 3 Department of Pharmaceutical Sciences, College of Pharmacy, University of Kentucky, Lexington, KY, USA

## Abstract

Abnormal dopaminergic transmission has been implicated as a risk determinant of HIV-1-associated neurocognitive disorders. HIV-1 Tat protein increases synaptic dopamine (DA) levels by directly inhibiting DA transporter (DAT) activity, ultimately leading to dopaminergic neuron damage. Through integrated computational modeling prediction and experimental validation, we identified that histidine547 on human DAT (hDAT) is critical for regulation of basal DA uptake and Tat-induced inhibition of DA transport. Compared to wild type hDAT (WT hDAT), mutation of histidine547 (H547A) displayed a 196% increase in DA uptake. Other substitutions of histidine547 showed that DA uptake was not altered in H547R but decreased by 99% in H547P and 60% in H547D, respectively. These mutants did not alter DAT surface expression or surface DAT binding sites. H547 mutants attenuated Tat-induced inhibition of DA transport observed in WT hDAT. H547A displays a differential sensitivity to PMA- or BIM-induced activation or inhibition of DAT function relative to WT hDAT, indicating a change in basal PKC activity in H547A. These findings demonstrate that histidine547 on hDAT plays a crucial role in stabilizing basal DA transport and Tat-DAT interaction. This study provides mechanistic insights into identifying targets on DAT for Tat binding and improving DAT-mediated dysfunction of DA transmission.

An estimated thirty-four million people worldwide are living with HIV. More than 50% of HIV-1 positive individuals suffer from neurological complications collectively referred to as HIV-1-associated neurocognitive disorders (HAND)^1^. HAND is a spectrum of disorders generally divided into three main groups: asymptomatic neurocognitive impairment (ANI; 33%), mild neurocognitive disorders (MND, 20–30%), and the more severe albeit rare HIV-associated dementia (HAD; 2–8%)^1^,^2^. Majority of HAND patients experience deficits in memory, concentration, and decision-making. HAND patients present neuropathological conditions that emerge from the continued exposure of the central nervous system (CNS) tissues to HIV-1, viral proteins, immune inflammation, and cART^3^,^4^. Currently, there are no promising therapeutic strategies for HAND. Considering the progressive and neurodegenerative nature of HAND, establishing an early intervention strategy would be beneficial to the preservation of neurocognitive function in HIV-infected individuals.

Converging lines of clinical observation, supported by imaging^5^,^6^, neuropsychological performance testing^7^,^8^, and postmortem examinations^9^, have implicated dopamine (DA) dysregulation with the abnormal neurocognitive function observed in HAND^10^,^11^. DA-rich brain regions (basal ganglia and related structures) are highly susceptible to the effects of both HIV infection and substance use. In the early stage of HIV infection, increased levels of DA and decreased DA turnover are found in the cerebrospinal fluid of therapy-naïve HIV patients with asymptomatic infection^12^, which may contribute to decreased levels of DA in DA-rich brain regions^8^,^13^,^14^ in the advanced stages of HIV infection. Importantly, HIV-induced elevated levels of extracellular DA in the CNS can stimulate viral replication in human macrophages within DA-rich brain regions^15^,^16^,^17^, resulting in viral protein release. It is commonly accepted that viral replication and proteins within the CNS are correlated with the persistence of HIV-related neuropathology and subsequent neurocognitive deficits^18^,^19^,^20^,^21^. Among HIV-1 viral proteins, transactivator of transcription (Tat) plays a crucial role in the neurotoxicity and cognitive impairment evident in neuroAIDS^3^,^22^. Tat can be detected in DA-rich brain areas^23^,^24^,^25^ and in the sera^26^,^27^ of HIV-1 infected patients. Long-term viral exposure can accelerate damage in the mesocorticolimbic DA system^10^,^28^,^29^ and to the brain pathways controlling motivation^30^,^31^,^32^. DA transporter (DAT)-mediated DA reuptake is critical for normal DA homeostasis. Human DAT (hDAT) activity is strikingly reduced in HIV-1-infected cocaine-using patients, correlating with the severity of HIV-1 associated cognitive deficits^5^,^6^. *In vitro*, the interplay of Tat and cocaine augments synaptic DA levels and Tat release by inhibiting DA transporter (DAT) activity^33^,^34^. By producing oxidative stress-induced damage to dopaminergic neurons, prolonged exposure to Tat protein eventually causes DAT-mediated dysregulation of DA to accelerate the progression of HAND^11^.

Through integrated computational modeling prediction and experimental validation, we have identified key residues in hDAT with which Tat interacts, which are critical for Tat-induced inhibition of DAT and transporter conformational transitions^35^,^36^,^37^. Our studies provide mechanistic insights into identifying residues on DAT for Tat binding, which allows the exploration of the molecular targets on DAT for therapeutic interventions, improving neurocognitive function of HAND. Our recent computational modeling study demonstrated that mutation of histidine to alanine at hDAT 547 (H547A) increases DA transport and attenuates Tat inhibitory effect on DAT function^38^. Basal DAT activity is regulated by several protein kinases and phosphatases^39^,^40^,^41^,^42^. Activation of protein kinase C (PKC) induces downregulation of DA reuptake^40^,^41^,^43^. In the present study, we pharmacologically determined the potential contribution of PKC phosphorylation to H547A-induced enhancement of DA transport by promoting or inhibiting of PKC activation. We also evaluated the functional influence of other substitutions of Histidine547 (His547) and its associated residues, tyrosine548 and tyrosine551, in DA transport as well as Tat-induced inhibition of DA uptake.

## Results

### Computational modeling: His547 and functional relevant residues of human DAT

**Figure 1:**
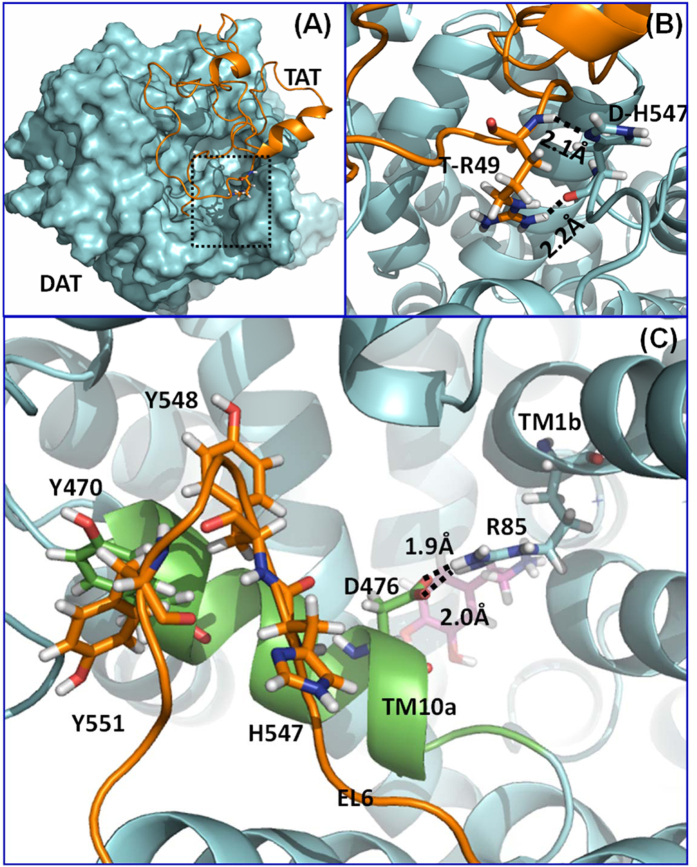
(**A**) Computational model of human dopamine transporter (hDAT) and HIV-1 Tat binding. hDAT and Tat are represented as cyan surface and golden ribbon, respectively. (**B**) A local view of DAT residue H547 and its direct interaction with Tat residue R49, hDAT is represented as a cyan ribbon. The residues are represented in sticks, with hydrogen bonds between the two residues represented with dashed lines labeled with their corresponding coordination distances. (**C**) Structural details of the residues Y470, Y551, H547, D476, and R85 on hDAT. hDAT is represented as a cyan ribbon, while the first part of transmembrane helix 10 (TM10a) and extracellular loop 6 (EL6) are colored in green and orange, respectively. Dopamine is represented as a ball-and-stick molecule in purple. Hydrogen bonds between D476 and R85 are indicated by dashed lines with coordinating distances labeled.

Based on the constructed hDAT-Tat binding model in our previous work^36^, both the side chain and backbone of H547 forms a hydrogen bond with residue R49 of HIV-Tat (Fig. 1B). The hydrogen bond with the H547 side chain is expected to be broken with the H547A mutation, which is consistent with decreased inhibitory activity of Tat on hDAT-H547A. Based on the computational model proposed in our previous work^36^, the Y548-Y470-Y551 interaction motif (denoted as the YYY motif) (Fig. 1C) could stabilize the first part of transmembrane helix 10 (TM10a) by tying down residue Y470 with extracellular loop 6. Instability of TM10a could promote the formation of a salt bridge between D476 (in TM10a) and R85 (in TM1b); however, the D476-R85 salt bridge would close the “entrance gate” of DA^35^,^36^,^44^,^45^,^46^,^47^. As a result, instability of TM10a is expected to block the DA entrance in the outward-open state, which may decrease the DA uptake efficiency of hDAT. It could be observed that the three phenol rings in the YYY motif are packed in a specific face to face style, which indicates that the EL6 should also take a specific conformation for the forming of the YYY motif. Therefore, any conformation change on EL6 that occurs close to the YYY motif is expected to regulate the interaction strength of the YYY motif. As one of the nearest residue to the YYY motif on EL6, residue H547 is an appropriate candidate for validating our proposed hypothesis. The H547P, H547A, H547D, and H547R single mutations were tested because proline and alanine mutations are expected to be the most significant change in backbone conformation, while aspartic acid or arginine mutation is expected to introduce significant side-chain conformational disturbances due to their non-neutral electrostatic potentials. According to the molecular dynamics (MD) simulation and the potential of main force (PMF) energy calculation performed in our previous work^36^, we found that the H547A mutation could enhance the strength of the YYY motif. On the contrary, the H547P mutation could weaken the strength of the YYY motif, which is consistent with the increased DA uptake efficiency for the H547A mutant and decreased DA uptake efficiency for the H547P mutant. Furthermore, the relatively weaker influence of the H547R and H547D mutations on DA uptake efficiency also support that the backbone conformation of residue 547 plays a key role in the stability of the YYY motif.

### Mutations of His547, Tyr548 and Tyr551 differentially influence DA uptake kinetics

**Figure 2:**
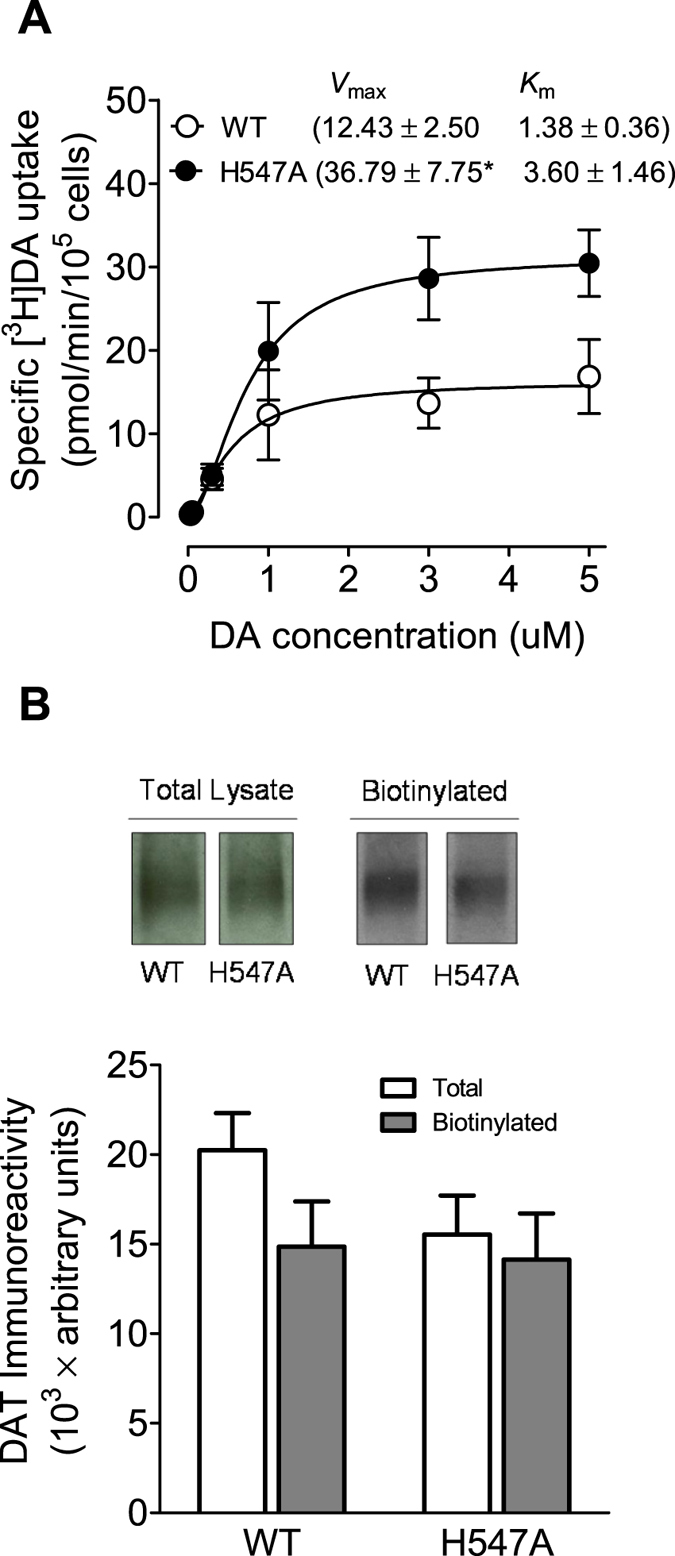
DA transport and DAT surface expression in WT hDAT and mutant. (**A**) Kinetic analysis of [^3^H]DA uptake in WT hDAT and H547A-hDAT. PC12 cells transfected with WT hDAT or H547A-hDAT were incubated with one of 6 mixed concentrations of [^3^H]DA as total rate of DA uptake. In parallel, nonspecific uptake of each concentration of [^3^H]DA (in the presence of 10 μM nomifensine, final concentration) was subtracted from total uptake to calculate DAT-mediated uptake. The *V*_max_ and *K*_m_ values were estimated by fitting the data to the Michaelis-Menten equation and represent the means from five independent experiments ± S.E.M. *p < 0.05 compared to control value (unpaired Student’s *t* test) (n = 5). (**B**) Cell surface expression of WT hDAT and H547A-hDAT was analyzed by biotinylation assay. Top panel: representative immunoblots (see supplemental information) PC12 cells expressing WT hDAT (WT) or H547A-hDAT (H547A) (n = 9).

**Figure 3:**
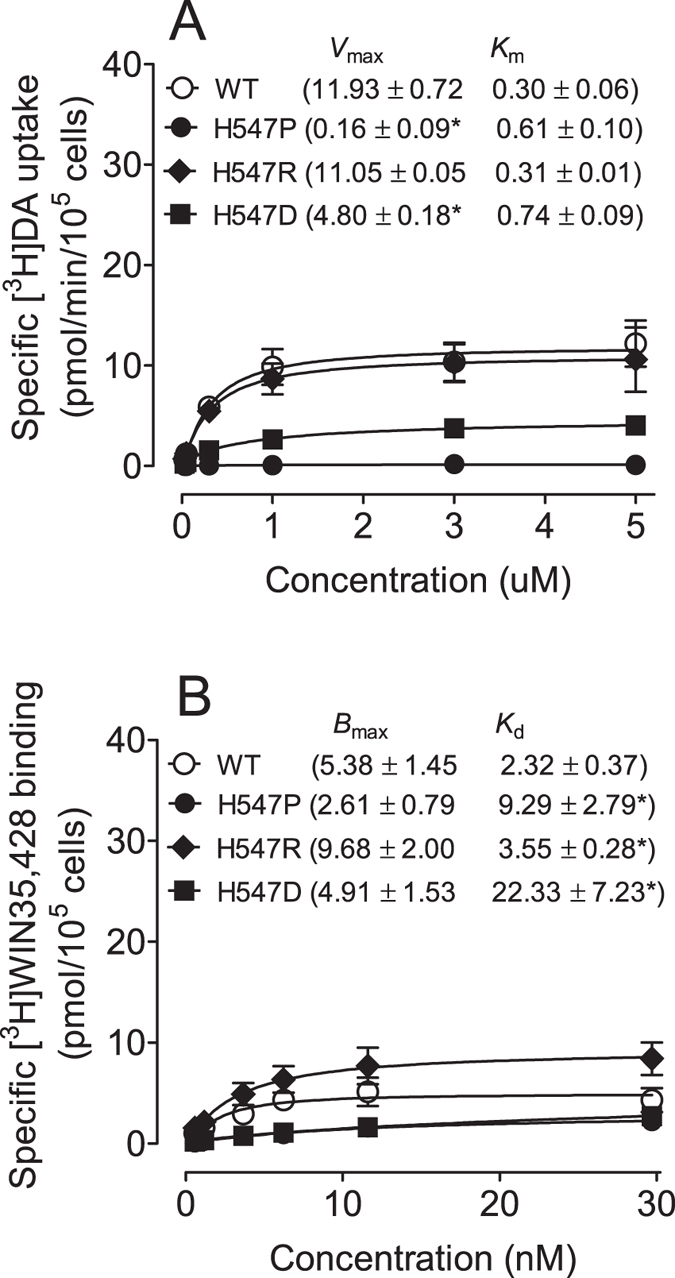
DA transport and DAT surface binding sites in WT hDAT and H547 substitutional mutants. (**A**) Kinetic analysis of [^3^H]DA uptake in WT hDAT and mutants. The *V*_max_ and *K*_m_ values were estimated by fitting the data to the Michaelis-Menten equation and represent the means from five independent experiments ± S.E.M. *p < 0.05 compared to WT hDAT value (unpaired Student’s t test) (n = 5). (**B**) Saturation binding of [^3^H]WIN35,428 in intact PC12 cells transfected with WT hDAT and mutants. The *B*_max_ and *K*_d_ values were estimated by fitting the data on a one-site binding curve and represent the means from four independent experiments ± S.E.M. *p < 0.05 compared to control value (unpaired Student’s *t* test) (n = 4).

To determine the functional influence of the His547 (H547A) mutation in DAT function, kinetic analysis of [^3^H]DA uptake was performed in PC12 cells transfected with WT hDAT or H547A-hDAT. As shown in Fig. 2A, compared to WT hDAT (12.43 ± 2.50 pmol/min/10^5^ cells), H547A-hDAT displayed a 196% increase in V_max_ value [36.79 ± 7.75 pmol/min/10^5^ cells, *t*_(8)_ = 2.99, *p* < 0.05, unpaired Student’s *t* test] without changes in K_m_ (H547A-hDAT, 3.60 ± 1.46 and WT hDAT, 1.38 ± 0.36 nM, *t*_(8)_ = 1.48, *p* = 0.08). To further determine whether other substitutions at His547 show differential effects on the basal DA transport, mutations at this residue (histidine to proline, arginine, and aspartate; H547P, H547R and H547D) were generated by site-directed mutagenesis. In separate experiments, the pharmacological profiles of [^3^H]DA uptake in PC12 cells transfected with WT hDAT or these mutants were determined. As illustrated in Fig. 3, compared to WT hDAT (11.93 ± 0.72 pmol/min/10^5^ cells), V_max_ values were not significantly changed in H547R-hDAT (11.05 ± 0.05 pmol/min/10^5^ cells), but dramatically decreased in H547P-hDAT [0.16 ± 0.09 nM, *t*_(6)_ = 6.95, *p* < 0.001] and H547D-hDAT [4.80 ± 0.18 nM, *t*_(6)_ = 4.24, *p* < 0.01], respectively. K_m_ values were not altered in H547P-, H547R-, and H547D-hDAT, compared to WT hDAT. Additionally, kinetic analysis of [^3^H]DA uptake was performed in PC12 cells transfected with WT hDAT, Y548H-hDAT, or Y551H-hDAT. As shown in Table 1, compared to WT hDAT (13.8 ± 2.8 pmol/min/10^5^ cells), the V_max_ values were decreased by 26% in Y548H-hDAT (10.2 ± 2.0 pmol/min/10^5^ cells) and 76% in Y551H-hDAT [3.3 ± 1.0, *t*_(6)_ = 3.53, *p* < 0.05]. On the contrary, there was no significant change in K_m_ in these mutants (Y548H-hDAT, 0.63 ± 0.1, Y551H-hDAT, 0.80 ± 0.3, and WT hDAT, 0.55 ± 0.1 μM).

To determine whether the H547A-induced increase in V_max_ is associated with altered subcellular distribution of DAT, biotinylation and immunoblot assays were performed. Three subcellular fractions were prepared from PC12 cells transfected with WT hDAT and H547A-hDAT. DAT immunoreactivity in both total fraction and cell surface fraction (biotinylated) were examined (Fig. 2B). No differences between WT hDAT and H547A-hDAT were found in the ratio of surface DAT (biotinylated DAT) to total DAT (biotinylated/total: WT hDAT, 0.75 ± 0.10; and H547A, 0.97 ± 0.17, *t*_(17)_ = 1.18, *p* = 0.25), indicating that increased V_max_ in H547A-hDAT is not due to alteration of the available DAT on the cell surface. With regard to H547P, neither surface nor total DAT signal was detectable (data not shown). The WIN35,428 binding site shares pharmacological identity with the DA uptake carrier^48^. To determine whether other substitutions on the His547 residue alter DA binding sites, kinetic analysis of [^3^H]WIN35,428 binding was performed in intact PC12 cells transfected with WT hDAT, H547P, H547R, or H547D. As shown in Fig. 3B, on comparison to WT hDAT, His547 mutants did not alter B_max_ values of [^3^H]WIN35,428 binding. However, K_d_ values were increased in H547P [9.29 ± 2.79 pmol/10^5^ cells, *t*_(6)_ = 2.48, *p* < 0.05], H547R [3.55 ± 0.28 pmol/10^5^ cells, *t*_(6)_ = 2.65, *p* < 0.05] and H547D [22.33 ± 7.23 pmol/10^5^ cells, *t*_(6)_ = 2.76, *p* < 0.05] relative to WT hDAT (2.32 ± 0.37 pmol/10^5^ cells).

### Mutations of His547, Tyr548 and Tyr551 differentially alter DA uptake inhibition potency of substrate and inhibitors

To determine whether mutations of His547, Tyr548, and Tyr551 influence selective binding sites on hDAT for DA, cocaine, and GBR12909, we tested the ability of substrate and DAT inhibitors to inhibit [^3^H]DA uptake in WT hDAT and its mutants (Table 2). In H547A, the apparent affinity (IC_50_) for DA was significantly decreased in H547A-hDAT [5356 ± 978 nM, *t*_(15)_ = 4.3, *p* < 0.05, unpaired Student’s *t* test] relative to WT hDAT (1720 ± 206 nM). There were no changes in the potencies of cocaine and GBR12909 for inhibiting [^3^H]DA uptake in H547A-hDAT compared to WT hDAT. We also tested whether H547A-hDAT alters the potencies of DA, cocaine, and GBR12909 for inhibiting [^3^H]WIN 35,428 binding. As shown in Supplemental Table 1, the IC_50_ value of cocaine for inhibiting DA uptake was decreased in H547A-hDAT [156 ± 36 nM, *t*_(8)_ = 2.70, *p* < 0.05] compared to WT hDAT (308 ± 55 nM). However, H547A-hDAT did not alter the potencies of DA and GBR12909 for inhibiting DA uptake. We determined whether H547P, H547R, and H547D differentially alter the potencies of DA, cocaine, and GBR12909 for inhibition of [^3^H]DA uptake (Table 2). The apparent affinity (IC_50_) for DA was significantly increased in H547P-hDAT [300 ± 49 nM, *t*_(15)_ = 4.30, *p* < 0.001], compared to the respective WT hDAT control (DA, 1720 ± 206 nM). No changes were observed in the potencies of cocaine and GBR12909 in H547P-, H547R-, and H547D-hDAT for inhibiting [^3^H]DA uptake compared to WT hDAT. In Y548H-hDAT and Y551H-hDAT, the affinity for DA was increased in Y551H [663 ± 70 nM, *t*_(17)_ = 2.60, *p* < 0.001] relative to WT hDAT (1720 ± 206 nM) while Y548H showed no change. In addition, the affinity for cocaine was increased in Y548H [77 ± 9 nM, *t*_(17)_ = 3.80, *p* < 0.01], and Y551H [59 ± 7 nM, *t*_(17)_ = 4.10, *p* < 0.001], respectively, relative to WT hDAT (294 ± 34 nM).

### Mutations of His547 attenuate Tat-induced inhibition of DA transport

**Figure 4:**
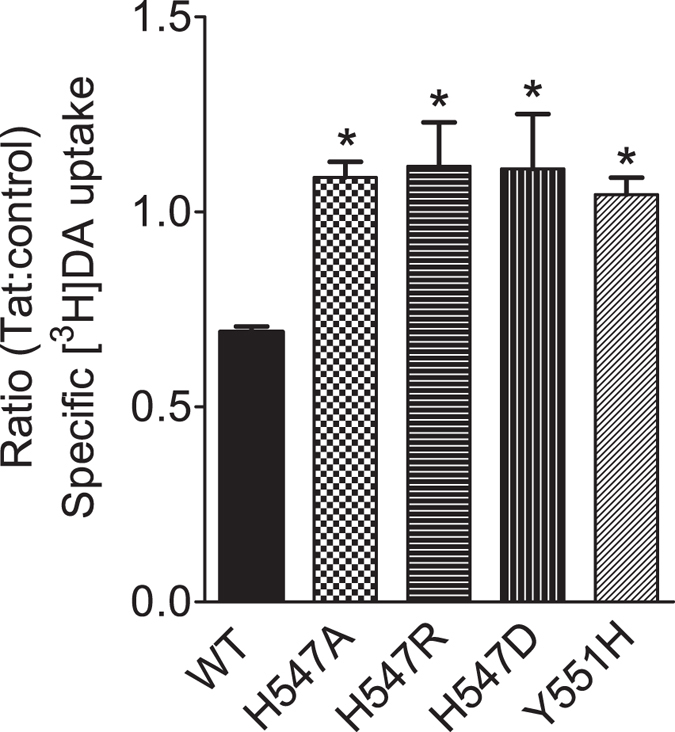
Effects of Tat on kinetic analysis of [^3^H]DA uptake in WT hDAT and His547 mutants. PC12 cells transfected with WT hDAT (WT), H547A-hDAT (H547A), H547R-hDAT (H547R), H547D-hDAT (H547D), or Y551H-hDAT (Y551H) were preincubated with or without recombinant Tat_1–86_ (rTat_1–86_) (140 nM, final concentration) at room temperature for 20 min followed by the addition of [^3^H]DA. Nonspecific uptake was determined in the presence of 10 μM final concentration of nomifensine. Data are expressed as the ratio of the specific [^3^H]DA uptake in the presence of Tat to that in the absence of Tat [in DPM: WT hDAT (Tat, 4794 ± 989 vs control, 6858 ± 1393); H547A (Tat, 6190 ± 1474 vs control, 5799 ± 1408); H547R (Tat, 5026 ± 1097 vs control, 4698 ± 1257); H547D (Tat, 2159 ± 557 vs control, 2021 ± 565); and Y551H (Tat, 662 ± 107 vs control, 642 ± 108)] n = 7–8. *p < 0.05 compared with WT hDAT control values.

Based on our computational prediction, it could be expected that mutations of His547 would eliminate a hydrogen bond between D-H547 and T-R49 (Fig. 1), impairing Tat binding on hDAT, thereby inducing an attenuation of Tat-induced inhibition of DA uptake. We examined the specific [^3^H]DA uptake in WT hDAT and the His547 mutants in the presence or absence of recombinant Tat_1–86_. Due to the difference in the specific [^3^H]DA uptake in WT hDAT and H547 mutants as shown in Figs 2 and 3, the inhibitory effect of Tat on DAT function in WT, H547A, H547P, H547R, H547D, and Y551H were presented as the ratio of Tat-mediated [^3^H]DA uptake to their respective controls (in the absence of Tat, Fig. 4). One way ANOVA revealed a significant main effect of genotype [F_(3, 28)_ = 5.72; *p* < 0.01]. Post hoc analysis showed that Tat (140 nM, final concentration) produced a 31% decrease in the specific [^3^H]DA uptake in WT hDAT relative to its control [in DPM: Tat (4794 ± 989) vs control (6858 ± 1393), *t*_(7)_ = 5.03, *p* < 0.01]; however, no effect of Tat on DA uptake was observed in H547A [in DPM: Tat (6190 ± 1474) vs control (5799 ± 1408)], H547R [in DPM: Tat (5026 ± 1097) vs control (4698 ± 1257)], H547D [in DPM: Tat (2159 ± 557) vs control (2021 ± 565)], and Y551H [in DPM: Tat (662 ± 107) vs control (642 ± 108)] (*p*s > 0.01, Bonferroni t-test), suggesting that mutations of H547 attenuate Tat-induced inhibition of DA uptake.

### Effects of H547A and H547D on Zinc regulation of DAT conformational transitions and basal DA efflux

**Figure 5:**
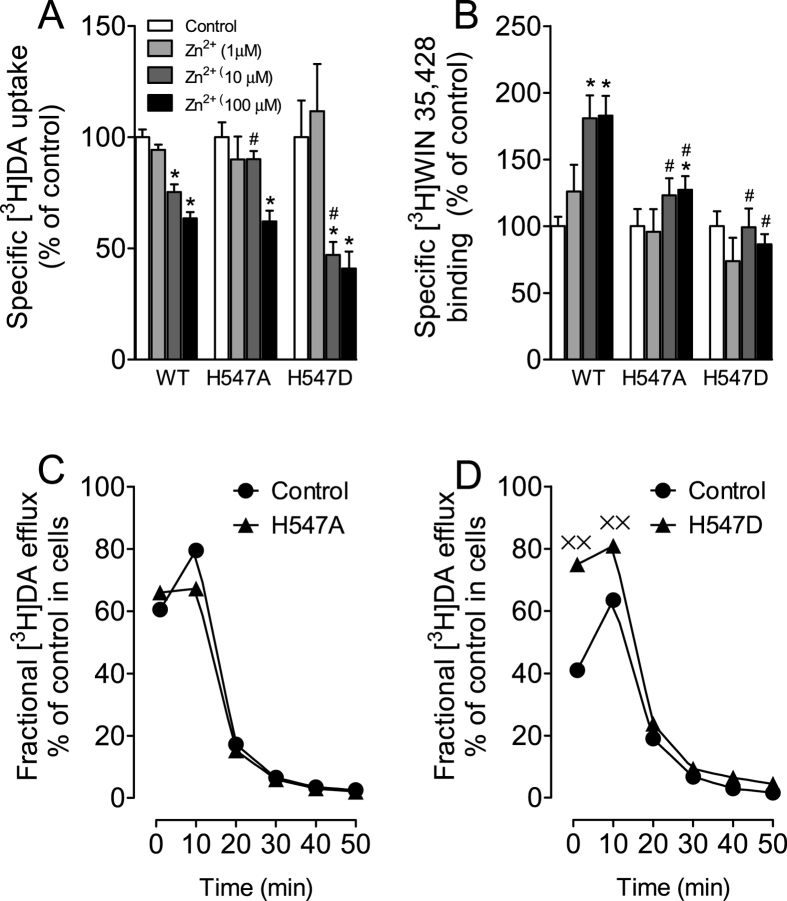
Effects of H547A and H547D mutants on transporter conformational transitions. Mutations of His547 affect zinc regulation of [^3^H]DA uptake (**A**) and [^3^H]WIN 35,428 binding (**B**). PC12 cells transfected with WT hDAT (WT), H547A-hDAT (H547A) and H547D-hDAT (H547D) were incubated with KRH buffer alone (control) or ZnCl_2_ (1, 10, 100 μM, final concentration) followed by [^3^H]DA uptake or [^3^H]WIN 35,428 binding (n = 5–7). The histogram shows [^3^H]DA uptake and [^3^H]WIN 35,428 binding expressed as mean ± S.E.M. of the respective controls set to 100% for the mutant. *p < 0.05 compared to control. ^#^*p* < 0.05 compared to WT hDAT with ZnCl_2_. Functional DA efflux of DA properties of H547A-hDAT (**C**) and H547D-hDAT (**D**) with their respective WT hDAT control. PC12 cells transfected with WT hDAT or mutants were preincubated with KRH buffer containing [^3^H]DA (0.05 μM, final concentration) at room temperature for 20 min. After incubation, cells were washed and incubated with fresh buffer as indicated time points. Subsequently, the buffer was removed from cells, and radioactivity in the buffer and remaining in the cells was counted. Each fractional efflux of [^3^H]DA in WT hDAT (WT) or mutants was expressed as percentage of total [^3^H]DA in the cells at the start of the experiment. Fractional [^3^H]DA efflux levels at 1, 10, 20, 30, 40 and 50 min are expressed as the percentage of total [^3^H]DA with preloading with 0.05 μM (WT hDAT: 13743 ± 3050 dpm, H547A-hDAT: 14464 ± 2547 dpm and H547D-hDAT: 1891 ± 428 dpm) presented in the cells at the start of the experiment (n = 4). ^××^*p* < 0.05 compared to WT hDAT (Bonferroni t-test).

We have demonstrated that Tat protein regulates DA transport allosterically^34^,^49^. To determine whether the His547 residue acts as a potential site for the allosteric modulation of hDAT-Tat interaction, we examined the effects of the respective mutations of His547 on Zn^2+^ modulation of [^3^H]DA uptake and [^3^H]WIN35,428 binding. In general, the conformational changes in DA transport processes involve conversions between outward- and inward-facing conformations^50^. Occupancy of the endogenous Zn^2+^ binding site in WT hDAT stabilizes the transporter in an outward-facing conformation, which allows DA to bind but inhibits its translocation, thereby decreasing DA uptake^51^ but increasing [^3^H]WIN35,428 binding^52^. Addition of Zn^2+^ is able to partially reverse an inward-facing state to an outward-facing state^51^,^52^. On the basis of this principle, the addition of Zn^2+^ to WT hDAT would inhibit DA uptake, whereas in a functional mutation in DAT Zn^2+^ might diminish the preference for the inward-facing conformation and thus enhance DA uptake. As shown in Fig. 5, two-way ANOVA on the specific [^3^H]DA uptake in WT and H547A-hDAT and H547D-hDAT revealed a significant main effect of zinc [F_(3, 48)_ = 51.1; *p* < 0.001] and a significant mutation × zinc interaction [F_(6, 48)_ = 4.90; *p* < 0.01]. The addition of Zn^2+^ significantly decreased [^3^H]DA uptake in WT and mutants in a zinc concentration-dependent manner (Fig. 5A). Compared to control (in absence of Zn^2+^), the addition of Zn^2+^ (100 μM) decreased [^3^H]DA uptake in WT (37%), H547A-hDAT (38%), and H547D-hDAT (60%), respectively (*ps* < 0.01, unpaired Student’s t test), suggesting these mutants do not affect the highest concentration of Zn^2+^-mediated regulation of DA transport. However, the addition of Zn^2+^ (10 μM) decreased [^3^H]DA uptake in WT (25%) and H547D-hDAT (53%), respectively (*ps* < 0.01, unpaired Student’s *t* test) but not in H547A-hDAT (10%), suggesting an attenuation of Zn^2+^-mediated regulation of DA transport by H547A-hDAT. In contrast, as shown in Fig. 5B, a two-way ANOVA on the specific [^3^H]WIN 35,428 binding in WT and H547A and H547D revealed a significant main effects of mutation [F_(2, 12)_ = 5.55; *p* < 0.05], zinc [F_(3, 36)_ = 19.19; *p* < 0.001], and a significant mutation × zinc interaction [F_(6, 36)_ = 7.76; *p* < 0.001]. The addition of Zn^2+^ (10 and 100 μM, final concentration) significantly increased [^3^H]WIN 35,428 binding in WT hDAT (10 μM, 80% and 100 μM, 82%) and H547A-hDAT (100 μM, 27%), respectively. The Zn^2+^ (10 and 100 μM)-induced increase in [^3^H]WIN 35,428 binding was significantly diminished in H547A-hDAT and H547D-hDAT.

To further determine the effects of His547 mutants on transporter conformational transitions, we examined the basal efflux levels of [^3^H]DA in WT hDAT, H547A-hDAT, and H547D-hDAT. With regard to H547A-hDAT (Fig. 5C), after preloading with 0.05 μM [^3^H]DA for 20 min at room temperature, PC12 cells transfected with WT hDAT and H547A-hDAT were washed and fractional DA efflux samples were collected at the indicated timepoints. Two-way ANOVA on the basal efflux of [^3^H]DA indicated a significant main effect of time [F_(5, 30)_ = 115.59; *p* < 0.001]. No significant main effects of mutation and mutation × time interaction were found. With regard to H547D-hDAT (Fig. 5D), a two-way ANOVA on the basal efflux of [^3^H]DA revealed significant main effects of mutation [F_(1, 8)_ = 17.38; *p* < 0.01] and time [F_(5, 40)_ = 252.09; *p* < 0.001] and mutation × time interaction [F_(5, 40)_ = 11.39; *p* < 0.001]. Post hoc analyses showed that compared to WT hDAT, DA efflux levels were elevated at 1 and 10 min in H547D-hDAT (*ps* < 0.05, Bonferroni t-test).

### Effects of H547A on basal PKC-mediated regulation of DAT function

**Figure 6:**
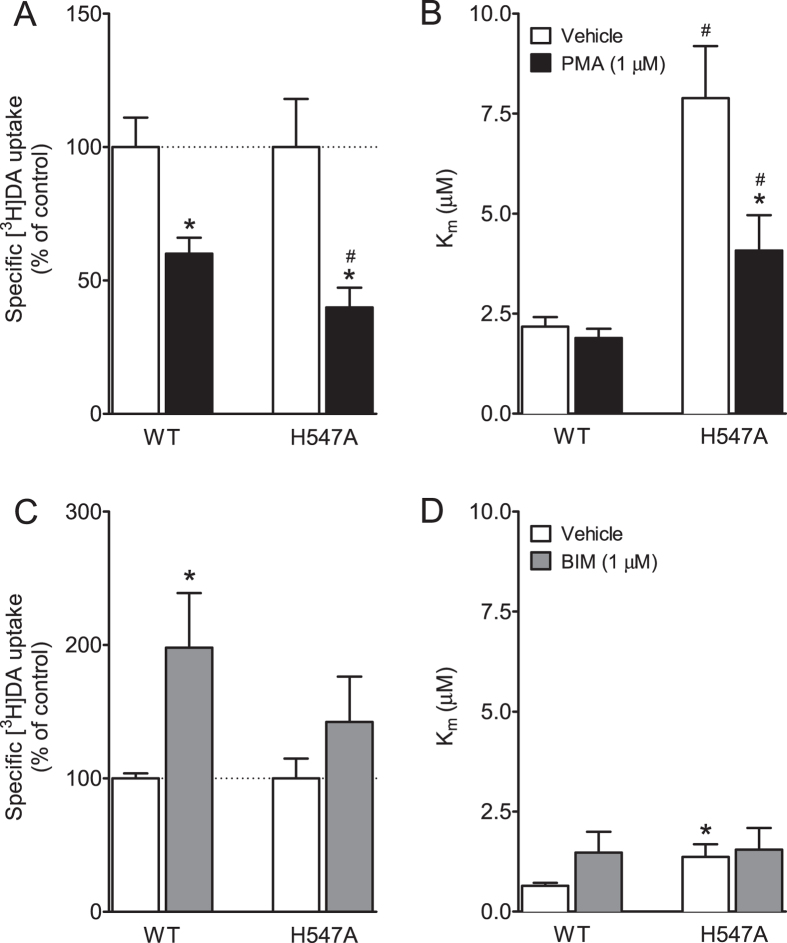
Effects of H547A on basal PKC-mediated regulation of DAT function. Kinetic analysis of [^3^H]DA uptake in PC12 cells transfected with WT hDAT or H547A-hDAT in the presence or absence of PKC activator PMA (1 μM) (**A**,**B**) or inhibitor BIM (1 μM) (**C**,**D**). *p < 0.05 compared to their respective controls. ^#^*p* < 0.05 compared to WT hDAT (n = 6–9).

To determine whether H547A-induced enhancement of V_max_ is associated with basal levels of DAT phosphorylation, kinetic analysis of [^3^H]DA uptake was performed in WT and H547A-hDAT in the presence or absence of a PKC activator phorbol 12-myristate 13-acetate (PMA). As shown in Fig. 6, a two-way ANOVA on the V_max_ values revealed significant main effects of mutation [F_(1, 32)_ = 14.67; *p* < 0.001] and PMA treatment [F_(1, 32)_ = 13.31; *p* < 0.001] and mutation × PMA treatment interaction [F_(1, 32)_ = 4.8; *p* < 0.05]. In the absence of PMA, the V_max_ [^3^H]DA uptake was higher in H547A-hDAT than WT [F_(1, 16)_ = 10.69; *p* < 0.01]. The addition of 1 μM PMA produced a 40% and 60% decrease in the V_max_ in WT and H547A-hDAT, respectively, compared to the respective control [F_(1, 16)_ = 4.43; *p* < 0.05]. With regard to the K_m_ values, two-way ANOVA revealed significant main effects of mutation [F_(1, 32)_ = 20.73; *p* < 0.001] and PMA treatment [F_(1, 32)_ = 7.36; *p* < 0.05]. No significant mutation × PMA treatment interaction [F_(1, 32)_ = 3.62; *p* = 0.066] was found. Post hoc tests showed that the K_m_ value was lower in WT hDAT (2.18 ± 0.24) than H547A-hDAT [7.89 ± 1.30, *t*_(15)_ = 4.06; *p* < 0.01] in the absence of PMA. After addition of PMA; the K_m_ value was still lower in WT hDAT (1.89 ± 0.23) than H547A-hDAT [4.08 ± 0.88, *t*_(15)_ = 4.06; *p* < 0.01], however, PMA significantly decreased K_m_ values in H547A-hDAT [4.08 ± 0.88 μM, *t*_(16)_ = 2.42; *p* < 0.05] but not in WT hDAT (*p* > 0.05) compared to the respective control.

In a separate experiment, we determined the specific [^3^H]DA uptake in the WT and H547 mutants in the presence or absence of a PKC inhibitor bisindolylmaleimide-I (BIM). As shown in Fig. 6(C,D), a two-way ANOVA on the V_max_ values revealed significant main effects of mutation [F_(1, 20)_ = 14.24; *p* < 0.01] and BIM treatment [F_(1, 20)_ = 4.55; *p* < 0.05]. No significant interaction of mutation × BIM treatment was observed. Addition of 1 μM BIM produced a 98% increase in the V_max_ in WT [F_(1, 10)_ = 6.96; *p* < 0.05] but not in H547A relative to their controls. The K_m_ value was lower in WT hDAT (0.65 ± 0.07) than H547A-hDAT [1.37 ± 0.31, *t*_(10)_ = 2.24; *p* < 0.05] in the absence of BIM. This difference was not observed between WT hDAT and H547A-hDAT after the addition of BIM.

## Discussion

Our recent computational-experimental study demonstrated that an alanine mutation to hDAT His547 (H547A) plays a crucial role in the hDAT-Tat binding and DA uptake by hDAT^38^. The present study aims to pharmacologically characterize His547 and its functional influence in Tat-induced inhibition of DA uptake. There were two main findings. First, H547A enhances DA transport in a PKC-dependent manner, and other substitutions of His547 (H547P, H547R, H547D) differentially altered DA uptake. Second, Tat inhibited DA uptake in WT hDAT, which was attenuated in His547 mutants. In addition, H547A attenuated zinc modulation of [^3^H]DA uptake and [^3^H]WIN35,428 binding, indicating that H547A leads to altered conformational transporter transitions. Overall, these results suggest potential therapeutic effects of targeting hDAT His547 on Tat-induced dysfunction of dopaminergic transmission observed in HAND patients.

Both His547 and the YYY motif (Tyr548-Tyr470-Tyr551) are found in the extracellular loop 6 (EL6) region of DAT. There was no significant change in the YYY motif structure unit during the conformational conversion of hDAT from the outward-open state to the outward-occluded state and then to the inward-open state, according to our computational simulation^38^. This observation suggests the possibility of promoting hDAT activity by increasing the stability of EL6 via enhancing the strength of the YYY motif^38^. The Y470H mutation is expected to destabilize the YYY motif by impairing both the Y470-Y548 and Y470-Y551 interactions, whereas the Y548H and Y551H mutations are expected to partially reproduce the effect of Y470H, as they only impair one of the Y470-Y548 and Y470-Y551 interactions. The Y470H mutant was reported to retain only ~18% (17.8%^37^,17.7%^38^ in our previous reports) V_max_ comparing to WT hDAT, whereas the present study shows that Y548H and Y551H mutants retain 24% and 74% V_max_ compared to WT hDAT, respectively (Table 1). Interestingly, based on the YYY motif model, the combined effect of the Y548H and Y551H mutations may be expected to retain 18% (24% × 74%) V_max_ compared to WT hDAT, which is consistent with the effect of the Y470H single mutation on V_max_. This observation confirms our computational prediction that YYY motif could stabilize the critical transmembrane 10 (TM10) by tying down Tyr470 with EL6 for enhanced transporter stability^38^. Disrupting this motif will result in perturbation to the transport function, as evidenced by the decrease in V_max_ in the mutants Y548H, Y470H, and Y551H. According to our previous work^35^,^36^,^37^, the center residue of YYY motif, i.e., residue Y470, is critical for Tat-induced inhibition of DA uptake, which indicates that stability of YYY motif is also involved in hDAT/Tat binding. Thus, mutating tyrosine551 to histidine may not only change the transporter’s capacity for uptake but also inflict a structural change of hDAT/Tat binding. Our results show that the Y551H mutation attenuates Tat-induced inhibition of DA uptake (Fig. 4), which is also consistent with our computational model.

To investigate the effect of the H547 mutation on influencing the YYY motif and hDAT transport, experiments were performed to obtain the kinetics data for four mutations, including H547A, H547P, H547R, and H547D. Alanine has the highest helix propensity; on the contrary, proline and glycine are generally known as “helix breakers”^53^,^54^. Therefore, the H547A and H547P mutations suggest two distinct directions of disturbing the backbone conformation. Additionally, mutation to other residues such as arginine or aspartic acid would introduce the most significant side-chain disturbance. Consequently, the H547R and H547D mutations would be reasonable probes for investigating the role of the side chain of residue 547. The present study shows that an alanine mutation on His547 increases the V_max_ by ~3-fold compared to WT hDAT, indicating that H547A is a critical residue that mediates the enhancement of DA transport, which supports our computational prediction^38^. Furthermore, the H547A-induced enhancement of DA uptake was not observed in other substitutions of H547, in which V_max_ was significantly decreased in H547P but not altered in H547R, whereas the effect of the H547D mutation on V_max_ decrease is relatively weaker than that of the H547P mutation. The hDAT activity shows higher sensitivity to backbone disturbances induced by H547A or H547P rather than side chain disturbances induced by H547D or H547R. Furthermore, the distinct helix propensity of H547A and H547P corresponds to the up-regulation and down-regulation of hDAT activity. Thus, the backbone conformation of residue His547 is expected to play an important role in hDAT function, which also supports our proposed hypothesis based on computational prediction^38^. Notably, the baseline K_m_ values for WT hDAT vary in the current study, which may be affected by the experimental conditions and individual experiments performed across different times. For example, the K_m_ values are relatively higher in either WT or H547A in Fig. 6B than others; this is because the PMA-mediated DA uptake assay was conducted at 37 °C. Previous studies have reported that DAT expression in plasma membrane is increased in a temperature-dependent manner^55^, which may cause increased V_max_ and decreased K_m_ for DA uptake. However, the K_m_ value of H547A hDAT was always compared to its respective WT hDAT control within each individual experiment. Importantly, our results demonstrate that H547A-hDAT displays increased K_m_ value for DA uptake relative to WT hDAT, indicating that mutating the His547 residue may alter the binding site of substrate DA. Given that DA transport efficiency by DAT is largely governed by surface DAT expression^56^, our results show that mutations of these residues do not alter either cell surface DAT expression or [^3^H]WIN35,428 binding sites in intact cells expressing these mutants, indicating that the altered V_max_ in these mutants is not due to changing surface DAT expression. Interestingly, mutant H547P displays a dramatic decrease in DA uptake, which is accompanied by decreased total DAT expression (data not shown). Considering that site-directed mutagenesis studies on DAT typically result in a decrease in DA uptake^35^,^37^,^57^,^58^, the enhancement of DA transporter as evidenced by the H547A mutant suggests that targeting this residue may provide an exciting knowledge basis for the development of novel concepts for the therapeutic treatment of the HAND.

Consistent with our previous reports^35^,^37^, the current results show that mutations of His547 completely eliminate the inhibitory effect of Tat on DA transport, further suggesting that the Tat molecule is associated with DAT through intermolecular electrostatic attractions and complementary hydrophobic interactions. Our computational study predicts that the side chain of H547 forms a hydrogen bond with residue R49 of HIV-Tat and that the alanine mutation of H547 would be expected to change the local backbone conformation of hDAT-H547 and eliminate the hydrogen bond between hDAT-H547A/Tat-R49 (Fig. 1). These findings indicate the important role of His547 in Tat-DAT interaction. The allosteric modulation of DAT is responsible for conformational transitions via substrate- and ligand-binding sites on DAT^50^,^59^. Given that Tat protein regulates DAT function allosterically^34^,^49^, we tested whether mutated His547 attenuates the Tat effect through alteration to a conformational transporter transition. First, our results show that H547A and H547D attenuate zinc-mediated decrease in DA uptake and increase in WIN binding. Second, H547D but not H547A enhances basal DA efflux, which further supports our previous report showing Tat protein induced enhancement of DA efflux in WT hDAT^60^. These results are consistent with our previous reports^35^,^37^, suggesting that disrupting the intermolecular interaction of Tat and DAT influences Tat-induced inhibition of DA uptake. Further understanding the functional relevance of additional residues in Tat on DAT modulation will provide useful feedback for further refining the computationally predicted binding model of DAT with Tat.

An important finding from the current study is that promoting PKC phosphorylation of DAT with PMA resulted in 40% and 60% reduction of DA uptake in WT hDAT and H547A, respectively. Similarly, preventing PKC phosphorylation of DAT with BIM produces a 98% and 42% increase in DA uptake in WT hDAT and H547A, respectively. This suggests a differential sensitivity to PMA- or BIM-induced activation or inhibition of DAT function between WT and H547A. It has been well characterized that PKC-dependent phosphorylation of DAT regulates DA uptake velocity^41^,^61^,^62^. One possibility is that mutation of His547 alters basal levels of PKC-mediated phosphorylation of DAT, thereby resulting in the enhanced DA uptake. Recent studies demonstrate that the serine-7 DAT residue is critical for PKC-dependent DAT phosphorylation^63^, and the alanine mutation of serine-7 results in an increase in DA uptake relative to WT DAT^64^. As the PKC phosphorylation sites on cytoplasmic domain (intracellular side) of hDAT are structurally far away from the residue H547 on the extracellular side of hDAT^37^,^38^, the H547A mutation is likely to regulate the PKC-mediated phosphorylation by allosteric effect. Therefore, future studies will be necessary to investigate the double mutant serine-7/His547 as an approach to identify whether the site of reduced PKC phosphorylation is on H547A-hDAT.

The current finding showing an unusual hDAT mutant capable of both enhancing DA transport and preventing Tat inhibitory effect on DAT is of general interest in therapeutic treatment of drug addiction. The interplay of Tat and cocaine augments synaptic DA levels and Tat release by inhibiting DAT activity^33^,^34^, which may contribute to the progression of HAND underlying the cognitive deficits in HIV-1 positive cocaine-using individuals^5^,^6^. Similarly, conditioned expression of Tat in the mouse brain further potentiates cocaine rewarding *in vivo*^65^. Together, these results suggest a synergistic effect of cocaine and Tat on DA transmission contributing to cognitive dysfunction and elevating the cocaine addictive effects. The current findings might provide novel insight into developing small molecule compounds that could bind to the unique residue on hDAT (His547), thereby not only preventing Tat interaction with DAT but also enhancing DA transport function. Ideally, the effectiveness of early intervention for HAND may combine such compound(s) with anti-retroviral therapy, which would be beneficial to the preservation of neurocognitive function in HIV-infected individuals.

## Methods

### Predicting the site for hDAT binding with Tat

The binding structure of hDAT with HIV-1 clade B type Tat was modeled and simulated based on the nuclear magnetic resonance (NMR) structures of Tat^66^ and the constructed structure of hDAT-DA complex. The protein-protein docking program ZDOCK^67^ was used to determine the initial binding structure of the hDAT-Tat complex. A total of 220,000 potential conformations were generated based on 11 NMR structures of Tat, then all of these conformations were evaluated and ranked by ZRANK^68^. Top-3,000 conformations selected from the protein-protein docking process were submitted to energy minimizations, and the docked structures were ranked according to the binding affinity estimated by using the Molecular Mechanics/Poisson-Boltzmann Surface Area (MM/PBSA) method^69^. Then the top-256 conformations were selected for further evaluation by performing molecular dynamics (MD) simulations. Based on the MD simulations, the most favorable hDAT-Tat binding mode (with the best geometric matching quality and reasonable interaction between hDAT and Tat) was identified, and the final hDAT-Tat binding structure was energy-minimized for analysis.

### Construction of plasmids

All point mutations of His547, Tyrosine548 (Tyr548), and Tyrosine551 (Tyr551) in hDAT were selected based on the predictions of the three-dimensional computational modeling and simulations. Based on the favorable hDAT-Tat binding mode (Fig. 1), it could be expected that mutations of His547 would eliminate a hydrogen bond between D-H547 and T-R49, which impair the binding affinity of Tat with hDAT, thereby diminishing Tat-induced inhibition of DA uptake. Mutations in hDAT at His547 [Histidine to Alanine (H547A), Proline (H547P), Arginine (H547R), or Aspartic acid (H547D)], Tyr548 (tyrosine to histidine, Y548H), and Tyr551 (tyrosine to histidine, Y551H) were generated based on wild type hDAT (WT hDAT) sequence (NCBI, cDNA clone MGC: 164608 IMAGE: 40146999) by site-directed mutagenesis. Synthetic cDNA encoding hDAT subcloned into pcDNA3.1+ (provided by Dr. Haley E Melikian, University of Massachusetts) was used as a template to generate mutants using QuikChange^TM^ site-directed mutagenesis Kit (Agilent Tech, Santa Clara CA). The sequence of the mutant construct was confirmed by DNA sequencing at University of South Carolina EnGenCore facility. Plasmid DNA were propagated and purified using a plasmid isolation kit (Qiagen, Valencia, CA, USA).

### Cell culture and DNA transfection

PC12 cells (ATCC^®^ CRL-1721^TM^, American Type Culture Collection, Manassas, VA) were maintained at 37 °C in a 5% CO_2_ incubator in Dulbecco’s modified eagle medium (DMEM, Life Technologies, Carlsbad, CA) supplemented with 15% horse serum, 2.5% bovine calf serum, 2 mM glutamine, and antibiotics (100 U/ml penicillin and 100 μg/mL streptomycin). Twenty four hours prior to transfection, cells were seeded into 24 well plates at a density of 1 × 10^5^ cells/cm^2^, or allowed to reach 100% confluence on plates. Cells were transfected with plasmids of WT hDAT or its mutants using Lipofectamine 2000 (Life Technologies, Carlsbad, CA). Twenty four hours after transfection, intact cells or cell suspensions were used for experiments.

### [^3^H]DA uptake assay

To determine whether DAT mutants alter DAT function, the maximal velocity (V_max_) or Michaelis-Menten constant (K_m_) of [^3^H]DA uptake were examined in PC12 cells transfected with WT hDAT or its mutants as previously reported^37^. Intact PC12 cells in 24-well plates were rinsed twice in Krebs-Ringer-HEPES (KRH) buffer (final concentration in mM: 125 NaCl, 5 KCl, 1.5 MgSO_4_, 1.25 CaCl_2_, 1.5 KH_2_PO_4_, 10 D-glucose, 25 HEPES, 0.1 EDTA, 0.1 pargyline, and 0.1 L-ascorbic acid; pH 7.4). The cells were then preincubated for 10 min at room temperature in KRH buffer with or without nomifensine for nonspecific binding (10 μM, final concentration). Next, the cells were incubated in KRH containing one of six concentrations of unlabeled DA (final DA concentrations, 0.03–5 μM) and a fixed concentration of [^3^H]DA (500,000 dpm/well, specific activity, 21.2 Ci/mmol; PerkinElmer Life and Analytical Sciences, Boston, MA) at room temperature for 8 min. Nonspecific uptake of each concentration of [^3^H]DA (in the presence of 10 μM nomifensine) was subtracted from total uptake to calculate specific DAT-mediated uptake.

To determine whether H547A-induced increase in V_max_ was mediated by a phosphorylation-dependent mechanism, kinetic analysis of [^3^H]DA uptake was measured in the presence or absence of PKC activator PMA (Tocris, Bristol, UK) or PKC inhibitor BIM (Sigma-Aldrich, St. Louis, MO). The concentrations were chosen based on previous reports^70^,^71^. In brief, intact PC12 cells transfected with WT or H547A-hDAT were preincubated with or without PMA or BIM (1 μM, final concentration) for 30 min at 37 °C or room temperature and then incubated with six concentrations of mixed [^3^H]DA for 8 min as described above. Nonspecific uptake of each concentration of [^3^H]DA (in the presence of 10 μM nomifensine) was subtracted from total uptake to calculate specific DAT-mediated uptake.

The competitive inhibition of DA uptake by DAT substrate and inhibitors was examined in intact PC12 cells transfected with WT hDAT or its mutants. Cells were preincubated in 400 μl KRH buffer containing 50 μl of one of a series of final concentrations of DA (1 nM–1 mM), GBR12909 (1 nM–10 μM), cocaine (1 nM–1 mM), or ZnCl_2_ (1, 10, 100 μM) for 10 min at room temperature and then incubated for 8 min after the addition of 50 μl of [^3^H]DA (0.05 μM, final concentration). The reaction for DA uptake in intact cells was terminated by removing reaction reagents and washing the cells twice with ice cold 1x KRH buffer. Cells were lysed in 500 μl of 1% SDS for an hour and radioactivity was measured using a liquid scintillation counter (model Tri-Carb 2900TR; PerkinElmer Life and Analytical Sciences, Waltham, MA). V_max_ and K_m_ were determined using Prism 5.0 (GraphPad Software Inc., San Diego, CA).

To determine the inhibitory effects of Tat on [^3^H]DA uptake, cells were dissociated with trypsin/EDTA (0.25%/0.1%, 1 mL for one 10 cm dish) and resuspended in culture medium at room temperature. After a 10-min incubation, the dissociated cells were harvested by centrifugation at 400 × g for 5 min at 4 °C and washed once with phosphate-buffered saline followed by another centrifugation at 400 × g for 5 min at 4 °C. The resulted cell pellets were resuspended in 1x KRH buffer. Specific [^3^H]DA uptake was determined in the cell suspensions prepared from WT hDAT and its mutants in the presence or absence of recombinant Tat_1–86_ (Diatheva, Fano, Italy; 140 nM, final concentration). Cell suspensions were preincubated with Tat for 20 min at room temperature and then incubated for 8 min after adding [^3^H]DA (0.05 μM, final concentration). Non-specific [^3^H]DA uptake was determined in the presence of 10 μM nomifensine. Incubation was terminated by immediate filtration through Whatman GF/B glass filters (presoaked with 1 mM pyrocatechol for 3 h). Filters were washed three times with 3 ml of ice-cold KRH buffer containing pyrocatechol using a Brandel cell harvester (model M-48; Brandel Inc., Gaithersburg, MD). Radioactivity was determined as described above.

### [^3^H]WIN 35,428 Binding Assay

Binding assays were conducted to determine whether mutated hDAT alters the kinetic parameters (B_max_ or K_d_) of [^3^H]WIN 35,428 binding in intact PC12 cells transfected with WT hDAT or mutants. Cells were washed with sucrose-phosphate buffer (final concentration in mM: 2.1 NaH_2_PO_4_, 7.3 Na_2_HPO_4_7H_2_O, and 320 sucrose, pH 7.4) and then incubated with one of the six concentrations of [^3^H]WIN 35,428 (84 Ci/mmol, PerkinElmer, 0.5–30 nM final concentrations) in a final volume of 500 μl on ice for 2 h. In parallel, nonspecific binding at each concentration of [^3^H]WIN 35,428 (in the presence of 30 μM cocaine, final concentration) was subtracted from total binding to calculate the specific binding. For the competitive inhibition experiment, assays were performed in duplicate in a final volume of 500 μl. Intact cells transfected with WT hDAT or its mutants were incubated in buffer containing 50 μl of [^3^H]WIN 35,428 (final concentration, 5 nM) and one of seven concentrations of unlabeled substrate DA (1 nM–100 μM), cocaine (1 nM–100 μM), GBR12909 (0.01 nM–1 μM) or ZnCl_2_ (1, 10, 100 μM) on ice for 2 h. Assays were terminated by removal of reaction reagents in well and then washed three times with ice-cold assay buffer. Cells were lysed with 1% SDS for an hour. Radioactivity was determined as described above.

### Cell surface Biotinylation

To determine whether DAT mutations alter DAT surface expression, biotinylation assays were performed as described previously^72^. PC12 cells transiently expressing hDAT or mutants were plated on 6-well plates at a density of 10^5^ cells/well. Cells were incubated with 1 ml of 1.5 mg/ml sulfo-NHS-SS biotin (Pierce, Rockford, IL) in PBS/Ca/Mg buffer (in mM: 138 NaCl, 2.7 KCl, 1.5 KH_2_PO_4_, 9.6 Na_2_HPO_4_, 1 MgCl_2_, 0.1 CaCl_2_, pH 7.3). After incubation, cells were washed 3 times with 1 ml of ice-cold 100 mM glycine in PBS/Ca/Mg buffer and incubated for 30 min at 4 °C in 100 mM glycine in PBS/Ca/Mg buffer. Cells were then washed 3 times with 1 ml of ice-cold PBS/Ca/Mg buffer and then lysed by addition of 500 ml of Lysis buffer (Triton X-100, 1 μg/ml aprotinin, 1 μg/ml leupeptin, 1 μM pepstatin, 250 μM phenylmethysulfonyl fluoride), followed by incubation and continuous shaking for 20 min at 4 °C. Cells were transferred to 1.5 ml tubes and centrifuged at 20,000 × g for 20 min. The resulting pellets were discarded, and 100 μl of the supernatants was stored at −20 °C for determination of immunoreactive total DAT. Remaining supernatants were incubated with continuous shaking in the presence of monomeric avidin beads in Triton X-100 buffer (100 μl/tube) for 1 h at room temperature. Samples were centrifuged subsequently at 17,000 × g for 4 min at 4 °C, and supernatants (containing the non-biotinylated, intracellular protein fraction) were stored at −20 °C. Resulting pellets containing the avidin-absorbed biotinylated proteins (cell-surface fraction) were resuspended in 1 ml of 1.0% Triton X-100 buffer and centrifuged at 17,000 × g for 4 min at 4 °C, and pellets were resuspended and centrifuged twice. Final pellets consisted of the biotinylated proteins adsorbed to monomeric avidin beads. Biotinylated proteins were eluted by incubating with 75 μl of Laemmli sample buffer for 20 min at room temperature and stored at −20 °C if further assays were not immediately conducted.

### Basal efflux assay

Basal DA efflux was performed at room temperature as described previously^35^,^37^. Intact PC12 cells transfected with WT hDAT or its mutants were preloaded with 0.05 μM [^3^H]DA for 20 min and then washed 3 times with KRH buffer prior to collecting fractional efflux samples. To obtain an estimate of the total amount of [^3^H]DA in the cells at the zero time point, cells from a set of wells (four wells/sample) were lysed rapidly in 1% SDS after preloading with [^3^H]DA. To collect factional efflux samples, buffer (500 μl) was added into a separate set of cell wells and transferred to scintillation vials after 1 min as an initial fractional efflux, and another 500 μl buffer was added to the same wells and collected after 10 min as second fractional efflux. Additional fractional efflux at 20, 30, 40, 50 min, respectively, was repeated under the same procedure. After last fractional efflux, cells were lysed and counted as total amount of [^3^H]DA remaining in the cells from each well.

### Data analysis

Results are presented as mean ± SEM, and *n* represents the number of independent experiments for each experiment group. Kinetic parameters (V_max,_ K_m_, B_max_, and K_d_) were determined from saturation curves by nonlinear regression analysis using a one-site model with variable slope. IC_50_ values for substrate and inhibitors inhibiting [^3^H]DA uptake or [^3^H]WIN 35,428 were determined from inhibition curves by nonlinear regression analysis using a one-site model with variable slope. For experiments involving comparisons between unpaired samples, unpaired Student’s *t* test was used to assess any difference in the kinetic parameters (V_max_, K_m_, B_max_, K_d_ or IC_50_) between WT and mutant; log-transformed values of IC_50_, K_m_ or K_d_ were used for the statistical comparisons. Significant differences between samples were analyzed with separate ANOVAs followed by post-hoc tests, as indicated in the results Section of each experiment. All statistical analyses were performed using IBM SPSS Statistics version 20, and differences were considered significant at *p* < 0.05.

## Additional Information

**How to cite this article**: Quizon, P. M. *et al*. Molecular mechanism: the human dopamine transporter histidine 547 regulates basal and HIV-1 Tat protein-inhibited dopamine transport. *Sci. Rep.* **6**, 39048; doi: 10.1038/srep39048 (2016).

**Publisher's note:** Springer Nature remains neutral with regard to jurisdictional claims in published maps and institutional affiliations.

## Supplementary Material

Supplementary Table 1

Supplementary Information

## Figures and Tables

**Table 1:** Kinetic properties of [^3^H]DA uptake in WT hDAT, Y548H-hDAT and Y551H-hDAT.

	V_max_ (pmol/min/10^5^ cells)	K_m_ (μM)
WT hDAT	13.8 ± 2.8	0.55 ± 0.1
Y548H	10.2 ± 2.0	0.63 ± 0.1
Y551H	3.3 ± 1.0*	0.80 ± 0.3

*p < 0.05 compared with WT hDAT.

**Table 2:** Summary of inhibitory activities in [^3^H]DA uptake assay in WT and mutated hDAT in the presence of DA, cocaine and GBR12909.

IC_50_ (nM)	WT hDAT	H547A	H547P	H547R	H547D	Y548H	Y551H
DA	1720 ± 206	5356 ± 978^*^	300 ± 48^*^	2454 ± 366	1168 ± 154	1744 ± 105	663 ± 70^*^
Cocaine	294 ± 34	338 ± 26	238 ± 26	470 ± 33	198 ± 32	77 ± 9^*^	59 ± 7^*^
GBR12909	262 ± 42	270 ± 85	332 ± 13	98 ± 17	344 ± 18	162 ± 44	279 ± 46

Data are presented as mean ± S.E.M. values from five to seven independent experiments performed in duplicates. *p < 0.05 compared with WT hDAT (unpaired Student’s *t* test).
