# Supplementary material for: Molecular mechanism: the human dopamine transporter histidine 547 regulates basal and HIV-1 Tat protein-inhibited dopamine transport
Source: Sci Rep. 2016 Dec 14;6:39048. doi: 10.1038/srep39048 (PMC5155291; doi:10.1038/srep39048)
Supplement: Supplementary Table 1 [file srep39048-s1.doc]

**Molecular mechanism: the human dopamine transporter histidine 547 regulates basal and HIV-1 Tat protein-inhibited dopamine transport**

Pamela M. Quizon^a^, Wei-Lun Sun^a^, Yaxia Yuan^b,c^, Narasimha M. Midde^a^, Chang-Guo Zhan^b,c^, and Jun Zhu^a*^

*^a^Department of Drug Discovery and Biomedical Sciences, South Carolina College of Pharmacy, University of South Carolina, Columbia, SC.*

*^b^Molecular Modeling and Biopharmaceutical Center, and ^c^Department of Pharmaceutical Sciences, College of Pharmacy, University of Kentucky, Lexington, KY*

Running title: *Histidine547 mutation on DAT*

*Corresponding Author:

Jun Zhu, MD., PhD

Department of Drug Discovery and Biomedical Sciences

South Carolina College of Pharmacy

University of South Carolina

715 Sumter Street, Columbia, SC 29208, USA.

Tel: +1-803-777-7924; Fax: +1-803-777-8356

E-mail: [zhuj@sccp.sc.edu](mailto:zhuj@sccp.sc.edu)

Supplemental Table 1 Summary of inhibitory activities in [^3^H]WIN 38,428 binding in WT-hDAT and mutated hDAT

|  | IC_50_ (nM) | | |
| --- | --- | --- | --- |
|  | DA | Cocaine | GBR12909 |
| WT hDAT | 1350 ± 382 | 308 ± 55 | 770 ± 193 |
| H547A-hDAT | 2416 ± 652 | 156 ± 36^*^ | 528 ± 120 |

* *p*<0.05 compared with WT hDAT (unpaired Student’s *t* test)
