# Supplementary material for: Molecular mechanism: the human dopamine transporter histidine 547 regulates basal and HIV-1 Tat protein-inhibited dopamine transport
Source: Sci Rep. 2016 Dec 14;6:39048. doi: 10.1038/srep39048 (PMC5155291; doi:10.1038/srep39048)
Supplement: Supplementary Information [file srep39048-s2.doc]

**Molecular mechanism: the human dopamine transporter histidine 547 regulates basal and HIV-1 Tat protein-inhibited dopamine transport**

Pamela M. Quizon^a^, Wei-Lun Sun^a^, Yaxia Yuan^b,c^, Narasimha M. Midde^a^, Chang-Guo Zhan^b,c^, and Jun Zhu^a*^

*^a^Department of Drug Discovery and Biomedical Sciences, South Carolina College of Pharmacy, University of South Carolina, Columbia, SC.*

*^b^Molecular Modeling and Biopharmaceutical Center, and ^c^Department of Pharmaceutical Sciences, College of Pharmacy, University of Kentucky, Lexington, KY*

*Corresponding Author:

Jun Zhu, MD., PhD

Department of Drug Discovery and Biomedical Sciences

South Carolina College of Pharmacy

University of South Carolina

715 Sumter Street, Columbia, SC 29208, USA.

Tel: +1-803-777-7924; Fax: +1-803-777-8356

E-mail: [zhuj@sccp.sc.edu](mailto:zhuj@sccp.sc.edu)

**Top left**. Surface (biotinylated) and intracellular DAT expression. Samples were loaded onto the gel and run under same experimental condition. Lanes indicate biotinylated protein (b, d, f, h) and intracellular protein (a, c, e, g). Lanes d and f were cropped and represented as surface DAT immunoblots for WT hDAT and H547A mutant, respectively, in Fig 2B (top panel).

**Lower left**. Total DAT expression. Samples were duplicated and loaded onto the gel and run under same experimental condition for WT hDAT (a, b), H547A (c, d), H547P (e, f), and D206L (g, f, unpublished data). Lanes b and d were cropped and represented as total DAT immunoblots for WT hDAT and H547A mutant, respectively, in Figure 2B (top panel).

**Blots on right**. The same blots shown in left were reprobed with β-tubulin antibody, which indicate an equal sample protein was loaded onto each lane.
